# Assembling Carbon Nanotube and Graphene in Chitosan/Sodium Alginate Hydrogels for Ion Removal Applications

**DOI:** 10.3390/polym17030353

**Published:** 2025-01-28

**Authors:** Sajad Paryav, Nariman Rajabifar, Amir Rostami, Mohsen Abbasi, Mohammad Akrami

**Affiliations:** 1 Department of Chemical Engineering, Persian Gulf University, Bushehr P.O. Box 75169-13817, Iran; sajjadparyav@gmail.com (S.P.); m.abbasi@pgu.ac.ir (M.A.); 2 Department of Polymer Engineering and Color Technology, Amirkabir University of Technology (Tehran Polytechnic), Tehran P.O. Box 15875-4413, Iran; nariman.rf@aut.ac.ir; 3 Department of Engineering, University of Exeter, Exeter EX4 4QF, UK

**Keywords:** hydrogel nanocomposites, adsorption kinetics, hydrogel crystallinity, chitosan hydrogel, water treatment, metal ions, sodium alginate

## Abstract

Hydrogels have emerged as a promising material for the removal of heavy metal ions from contaminated water owing to their high water absorption capacity and biocompatibility. Despite notable advancements in improving the adsorptive capacity of hydrogels, the demand for a more efficient structure persists. Here, we explore the ion adsorption performance of crosslinked hydrogels based on chitosan and sodium alginate with various ratios of carbon nanotubes (CNT) and graphene platelets (GNP). This study highlights the adsorption of chromium ions and the thermal stability of hydrogels for pure, single-particle, and hybrid nanocomposites. The results depict a uniform microstructure attained when CNT, GNP, or both are implemented into the hydrogel due to the strong interaction of functional moieties. The incorporation of CNT and GNP manipulates the crystalline structure of the hydrogels, lowering their crystallinity by around 28% and 13%, respectively. The synergistic effect of CNT and GNP in hybrid hydrogels raises the decomposition temperature by 16%, indicating a favorable interplay interaction between nanoparticles and polymers. Calculations of the adsorption capacity accentuate such a mutual effect between CNT and GNP in various loads of ion capture from aqueous solutions. Kinetic models fitted to the hydrogel nanocomposites reveal that the pseudo-second-order model aligns better with the experimental data in comparison to the pseudo-first-order and intraparticle diffusion models, addressing the adsorption mechanisms while capturing chromium ions.

## 1. Introduction

The escalating activities of industries have led to alarming levels of heavy metal emission release, posing notable threats to human health and the environment by polluting waterways [1,2]. Metallic ions, such as hydrogen chromate (HCrO_4_^−^) and chromate (CrO_4_^2−^), have adverse effects on the body through pulmonary congestion, skin lesions, and immune system suppression [3,4]. They can also cross cellular membranes and lead to the damage of proteins, generating oxidative stress and causing DNA mutations, thereby disrupting cellular processes. Although an array of methods have been developed to eliminate metal ion residues in water, including ion exchange, membrane filtration, and coagulation-flocculation, adsorption emerges as a viable approach owing to its affordability, high efficiency, and convenience in design and application [5,6,7].

Hydrogels are ideal materials for sorption applications since they capture and retain large amounts of water without sacrificing their structural properties, providing effective performance in scavenging metallic ions [8,9,10]. Endowed with high surface areas and numerous adsorption sites available, the porous three-dimensional structure and polar functional groups render these materials suitable for ion removal applications from aqueous solutions. Additionally, hydrogels are non-toxic and biocompatible due to their hydrophilic nature and are, thus, well-suited for environmental remediation applications [11]. The sustainability of hydrogels can be boosted through the implementation of biodegradable polymers, such as chitosan (CS) and sodium alginate (SA), leading to the reduction of the residual metal ion risk, cutting costly regeneration or disposal processes, and improving ion binding capacities and selectivity [12,13,14]. For instance, CS/SA hydrogels have been shown to remove chromium ions with a 75% efficiency over three adsorption/desorption cycles [15]. This performance can further reach beyond 98% if the nickel nanoparticles are doped within the hydrogel pores.

The integration of nanomaterials into hydrogels is viewed by many as a suitable approach to enhance ion-capturing efficiency since the high surface area of nanoparticles synergizes with hydrogel porosity, leading to improvement of the applicability of hydrogels [16,17,18,19]. Carbonic nanoparticles, such as carbon nanotubes (CNT) and graphene nanoplatelets (GNP), are prominently utilized in the sorption processes and as reinforcements in hydrogels due to their exceptionally high surface area, facile functionalization capabilities, and inherent biocompatibility [20,21,22]. Eldeeb et al. [23] showed the ion-capturing improvement of hydrogel nanocomposites based on CS and polyvinyl alcohol (PVA) by inducing CNT. Using the Langmuir model, they reported 217.4 mg/g as the maximum adsorption of chromium ions in CS/CNT/PVA hydrogels compared to other sorbents. An optimal pH of 1.5 was also observed to facilitate maximum ion adsorption, beyond which a decline in adsorption efficiency was evident with increasing pH values, exhibiting a downward trend. Furthermore, the adsorption kinetics conformed to a pseudo-second-order model, suggesting that the adsorption process was predominantly governed by chemical adsorption mechanisms. They involved the formation of chemical bonds through the sharing/exchange of electrons between the adsorbent and adsorbate, indicative of strong valence forces. By integrating graphene oxide (GO) nanoparticles in CS hydrogels, Zhao et al. [24] also showed that the adsorption process follows the pseudo-second-order model. They justified the high adsorption efficiency of the hydrogel nanocomposites with the three-dimensional network formed by the self-assembly of GO sheets and CS molecules. The CS/GO hydrogel nanocomposite was found to possess enhanced thermal stability, owing to the formation of crosslinked nanostructures during synthesis. The crosslinking process had an insignificant effect on the GO sheets, retaining their adsorption capabilities. In another work, the adsorption efficacy of the CS/GO hydrogel nanocomposites was evaluated on individual solutions containing copper, zinc, and nickel ions [25]. For all solutions, the results revealed a remarkable performance of the hydrogels to capture the contaminants. This was attributed to the combined effects of CS’s natural adsorption properties and the large surface area and functionality of GO.

In this work, we investigate the performance of ion adsorption in SA/CS hydrogels with the singular and hybrid assembling of CNT and GNP. The structure-property relation between nanoparticles and crosslinked hydrogels is discussed to unveil the quality of interaction among elements. By employing X-ray analysis on the SA/CS hydrogel nanocomposites, we demonstrate that nanoparticles can manipulate the crystallinity of the hydrogels. The kinetics of chromium ion adsorption into pure SA/CS hydrogels and hybrid nanocomposite hydrogels is fitted to the experimental data using pseudo-first-order, pseudo-second-order, Elovich, and intraparticle diffusion models

## 2. Experimental

### 2.1. Materials

Chitosan (CS), with a molecular weight of 120 kDa (deacetylation degree ≈ 85%), and sodium alginate (SA), with a molecular weight of 80 kDa (polymerization degree: 400–600), were purchased from Sigma Aldrich (Burlington, MA, USA). GNP (N002-PDR) composed of one to three monolayer graphene sheet stacks (surface area: 400–800 m^2^/g, thickness < 1 nm, $d_{ave}<10\;\mu m$) was obtained from Angstron Materials (Dayton, OH, USA). CNT (NC-7000, ≈9.5 nm in diameter, >95%), with a surface area of 250–300 m^2^/g, was purchased from Nanocyl (Sambreville, Belgium). Other chemicals, including acetic acid, K_2_CrO_4_, NH_3_, and CaCl_2_ (>99%), were purchased from Merck (Darmstadt, Germany). All ingredients were used without further purification unless noted otherwise.

### 2.2. Synthesis of Polymer Hydrogels and Hydrogel Nanocomposites

For a pure hydrogel, 4 g of SA was added to 100 mL of distilled H_2_O in a beaker containing a magnetic stir bar. The mixture was stirred for 2 h and heated at 85 °C until it reached a uniform solution. In a different beaker, 3 g of CS was added to 97 mL of distilled H_2_O with the same setup. A total of 3 mL of acetic acid was added to the CS solution while vigorously stirring. The CS solution was then added to the aqueous SA, followed by 1 more hour of mixing. The SA/CS was homogenized twice using a Daihan high shear homogenizer for 10 min at 10,000 rpm and a 15 min interval. Subsequently, the SA/CS solution was added to 0.1 mol/L CaCl_2_ to induce chemical crosslinking and rested overnight. After thoroughly rinsing them with distilled H_2_O to remove any unreacted chemicals at pH ≈ 7, the hydrogels were dried for at least 48 h under ambient conditions. For a typical synthesis of hydrogel nanocomposite, CNT and GNP were added to the SA/CS solution and mixed for 4 h at 85 °C using a magnetic stirrer. The single-particle hydrogel nanocomposites contained 3 wt% of either CNT or GNP. The hybrid hydrogel nanocomposites were prepared with proportions of 3:1, 1:1, and 1:3. The nanocomposite suspension was homogenized in identical settings to those previously employed, followed by a high-intensity sonication in an ultrasonic processor (Bandelin, Berlin, Germany, 35 kHz, 400 W) for 15 min. The obtained suspension underwent another stirring for 1 h as well as homogenization to minimize agglomeration and stacks of nanoparticles. The hydrogel nanocomposites were obtained after crosslinking and drying.

### 2.3. Characterization

Scanning electron microscopy (SEM) images were obtained using a TESCAN MIRA III (Czech) with an accelerating voltage of 30 kV. X-ray diffraction (XRD) data were acquired using a PHILIPS PW1730 (Netherlands) instrument with CuKα radiation (λ=1.54056 nm) at a generator voltage of 40 kV with a scanning speed of 5° per minute under room conditions. The thermogravimetric analysis (TGA) was performed using a Perkin Elmer (Shelton, CT, USA) TA instrument under nitrogen gas and a heating rate of 10 °C/min. Fourier transform infrared (FTIR) spectra were recorded on a JASCO (Tokyo, Japan) instrument in the wave number range of 4000–400 cm^−1^ with a resolution of 4 cm^−1^. The chromium ion adsorption was measured using a Secomam UV-vis spectrometer by dispersing 10 mg of pure hydrogel and hybrid hydrogel nanocomposites in distilled H_2_O. The batch adsorption method was performed in flasks containing 2 g of the hydrogels and 100 mL of chromium solution with a concentration of 50 mg/L and a pH level of 5. The flasks containing the hydrogel beads were placed in a constant-temperature bath oscillator, where they were vibrated under ambient conditions. Following an 8 h contact time, the beads were separated from the solution using a sieve and tray. The initial and final concentrations of chromium were then measured using UV-visible spectrophotometry.

## 3. Results and Discussion

### 3.1. Microstructure of Hydrogel Nanocomposites

**Figure 1:**
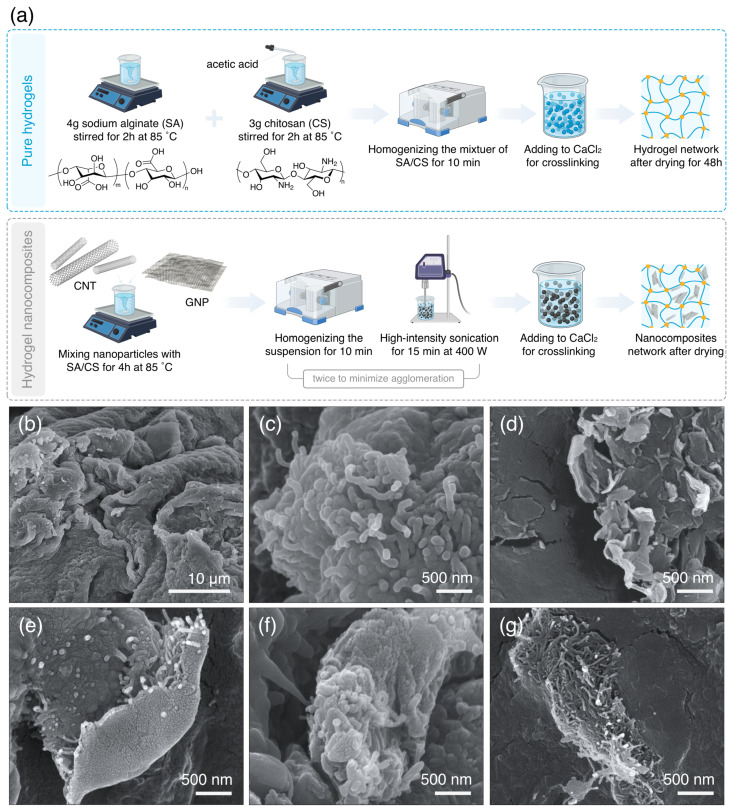
Microstructure formation of hydrogel nanocomposites. (**a**) Schematic of materials and structure development of SA/CS hydrogels. SEM micrographs of hydrogels: (**b**) crosslinked SA/CS hydrogel, (**c**) SA/CS/3%CNT, (**d**) SA/CS/3%GNP, (**e**) SA/CS/CNT:GNP (1:3), (**f**) SA/CS/CNT:GNP (1:1), and (**g**) SA/CS/CNT:GNP (3:1).

The schematic and SEM image of crosslinked hydrogel reveal a porous structure, as illustrated in Figure 1a,b. The presence of pores on the hydrogel surface, as evidenced by such a rough surface morphology, provides a clear indication of metal adsorption. The native porosity of hydrogel indeed promotes molecular interactions and enhances its adsorption capacity because of the high surface area and binding sites available within the pores [26]. Notably, the dispersion of single-particle nanomaterial, including CNT and GNP, was homogeneous throughout the hydrogel matrix due to the low work of adhesion between the hydrogel and the nanomaterials, therefore preventing aggregation (Figure 1c,d). Such a favorable tendency among the components was also observed in hybrid nanocomposites, confirming effective hydrogel synthesis (Figure 1e–g). We hypothesize that the uniform dispersion of dual-assembled nanoparticles can be attributed to the distinct structural differences between the two nanoparticle types, leading to minimal inter-particle interactions and, thereby, facilitating a homogeneous distribution. As the CS/SA hydrogel structure uniformly encompassed the hybrid CNT and GNP, we conclude the formation of a large contact surface area, thus improving the properties significantly. Such a strong three-dimensional network among nanoparticles, hydrogel-nanoparticles, and hydrogel chains demonstrates robust interfacial adhesion between elements, leading to a reinforced porous structure.

**Figure 2:**
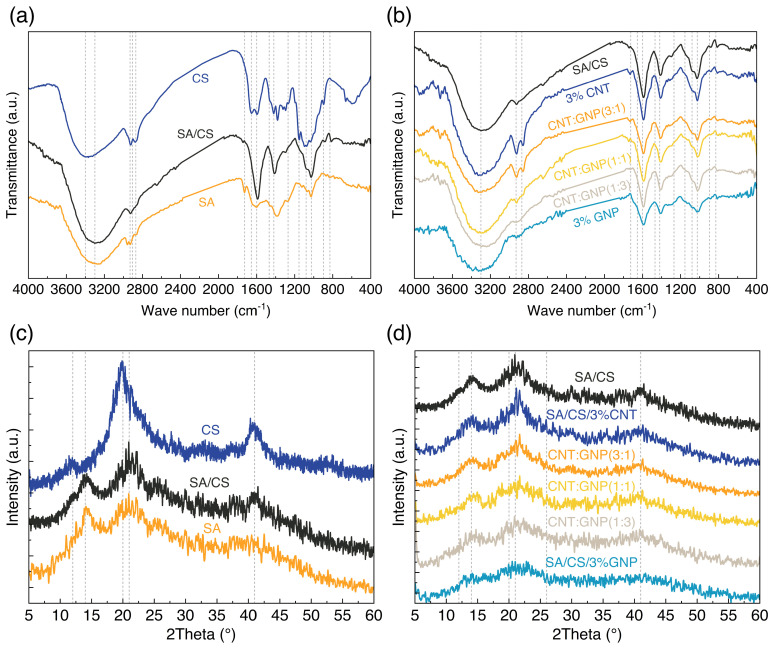
Structural analysis of the hydrogels. (**a**) FTIR spectra of neat polymers and the SA/CS hydrogel. (**b**) FTIR spectra of the SA/CS hydrogel and its nanocomposites. (**c**) XRD spectra of neat polymers and the SA/CS hydrogel. (**d**) XRD spectra of the SA/CS hydrogel and its nanocomposites.

To confirm the intramolecular connections and interaction between the hydrogel and the nanoparticles, the FTIR analysis was employed on the samples, as shown in Figure 2a,b. The peaks observed in the SA/CS spectrum are a combination of the peaks observed for CS and SA. In particular, the broad peaks between 3600 and 3000 cm^−1^ indicate hydroxyl and stretching vibrations of N-H groups in the pure CS spectrum. The peaks at 1595–1408 cm^−1^ are related to the stretching vibration of symmetric and asymmetric -COO- bonds. Also, the amide III bond, secondary, and primary amine groups are separately located at 1348, 1598, and 1656 cm^−1^ wavelengths. For the pure SA powder, broad peaks between 3500 and 3000 cm^−1^ indicate stretching vibrations of O-H groups and stretching vibrations of N-H moieties. Aliphatic C-H stretching vibrations are observed in the wavenumber range of 2920–2850 cm^−1^. The observed bonds in the wave number range of 1609 and 1390 cm^−1^ are also attributed to the asymmetric and symmetric vibrations of the carboxylate ion, respectively. Looking at the SA/CS spectrum, the broad peaks between 3500 and 3000 cm^−1^ belong to the stretching vibrations of O-H and N-H groups. Some peaks slightly shifted, and the intensity of other peaks increased due to the formation of hydrogen bonds between CS and SA functional groups. Such observations in the FTIR results are in favor of the previous studies showing an effective interaction among components [27].

The effective interaction among constituents can be further unveiled with the XRD spectra of the CS, SA, and SA/CS hydrogel, as shown in Figure 2c. Accordingly, CS features three peaks at the angles of 12°, 20°, and 42°, while the two peaks at the angles of 14° and 21° belong to SA. The combination of the spectra of the polymers is evident in the SA/CS hydrogel upon three given peaks at the angles of 14°, 21°, and 42°. Figure 2d illustrates the results of the XRD test for the SA/CS hydrogel, single-particle SA/CS hydrogel nanocomposites, and hybrid nanocomposites with CNT and GNP. The presence of similar peaks in the graphs exhibits the fingerprint of CS and SA in all samples. The intensity of the peak at 2θ=20° for the SA/CS hydrogel declines with the incorporation of nanoparticles due to their high concentration inhibitory effect on crystallization.

According to the computed crystallinity index from the XRD spectra in Figure 2, CS exhibits a higher crystalline content not only due to its high molecular weight compared to SA but also its longer chain length that facilitates more extensive interchain hydrogen bonding. SA, on the other hand, has a more rigid and extended chain conformation due to electrostatic repulsion, leading to less Brownian motion required to create ordered structures [28,29,30]. The spectrum of the SA/CS hydrogel is consequently located between those of the two neat polymers. The incorporation of CNT into the hydrogel matrix results in a significant reduction of the crystallinity index by ca. 28%. This occurrence suggests that physical confinement induced by CNT disrupts polymer-polymer interactions, thereby hindering crystal growth within the hydrogel [31]. In particular, the addition of CNT promotes robust interactions between the nanotubes and the hydroxyl and carboxyl moieties present in both SA and CS, facilitated by the high surface area of the polymers and nanoparticles. The crystallinity of polymers is compromised by CNT, resulting in a diminution of crystallite sizes, as quantified in Table 1 via the Scherrer equation. The layered structure of GNP accentuates the physical confinement even further, thereby potentiating a more pronounced disruption of SA/CS crystal growth and accompaniment reduction in crystallite sizes.

The component ratio modulates the crystallinity index of hybrid nanocomposite hydrogels. The SA/CS/CNT:GNP (3:1) nanocomposite depicts a crystallinity index of ca. 17.6% at 31%, surpassing its single-particle counterparts and the neat crosslinked hydrogel (SA/CS) by a significant margin. This enhancement can be attributed to the long, wavy nanotubes, which not only bridge the layered GNP but also preclude the reaggregation of GNP nanolayers, thereby mitigating the clumping. Similarly, a comparative analysis of SA/CS/CNT:GNP (1:3) and a single GNP-loaded SA/CS hydrogel reveals that the nanotubes facilitate the scattering of stacked GNP layers, fostering an intimate interaction with the polymer matrix and culminating in improved crystallinity. We should note that the re-aggregation of graphene sheets during the synthesis process, driven by van der Waals forces and strong π-π interactions, is typically indicated by a characteristic peak at around 26.4 degrees [32,33]. However, in the case of nanocomposite hydrogels containing 3% GNP as well as hybrid samples, the absence of peaks in the 26-degree range suggests that graphene re-aggregation is successfully mitigated. This outcome can be attributed to the efficacy of the synthesis and the robust interfacial adhesion between GNP and the hydrogel, both of which, collectively, prevent sheet re-aggregation and facilitate a homogeneous dispersion.

### 3.2. Thermal Stability of Hydrogel Nanocomposites

**Figure 3:**
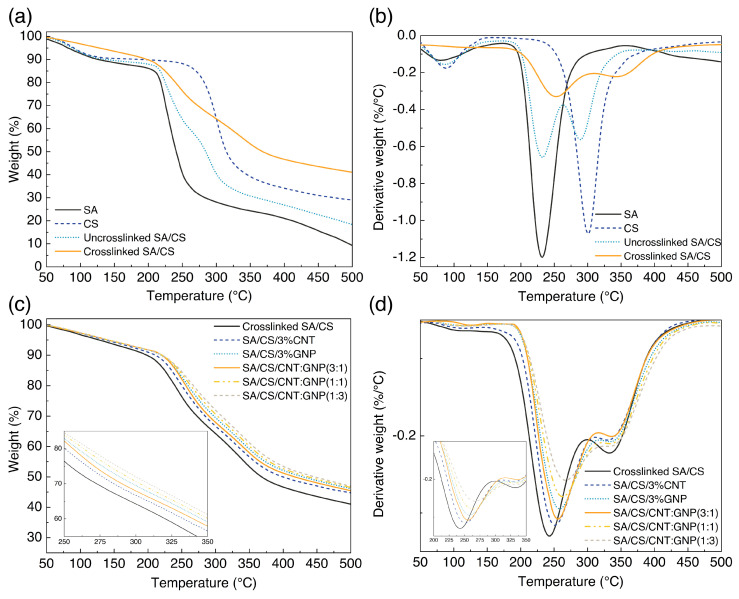
The thermal analysis of SA/CS hydrogel nanocomposites. (**a**) TGA curves of pure SA/CS hydrogels, SA, and CS. (**b**) DTG curves of pure SA/CS hydrogels and neat polymers. (**c**) TGA curves of SA/CS hydrogel nanocomposites with CNT and GNP. (**d**) DTG curves of SA/CS hydrogel nanocomposites with CNT and GNP.

Preserving hydrogel pore size and swelling capacity is vital for withstanding thermal fluctuations during desorption and regeneration, sustaining its structure and functionality. We examined the thermal stability of pure hydrogels and their hybrid nanocomposites to gain a better understanding of such behavior. The results of TGA and its derivative (DTG) curves on the individual element used during synthesis, SA/CS hydrogels, and crosslinked hydrogels are illustrated in Figure 3a,b. As seen, pure SA/CS hydrogel undergoes a two-stage degradation process when exposed to heat, with the initial stage occurring between 50 and 150 °C and the subsequent one taking place within the temperature range of 200–400 °C. During the initial part, the weight loss is primarily attributed to the evaporation of water and other small molecules, whereas the second stage is characterized by a significant weight loss due to the degradation and breakdown of the polymer backbone.

Looking at the TGA-DTG graphs of the constituents, it is evident that CS features more thermal stability on account of a lower weight loss at higher temperature ranges compared to SA. This behavior is endowed with the higher crystalline content of CS along with the molecular weight, as already mentioned. The thermal behavior of the pure hydrogel falls, therefore, within the range of CS and SA, since they are the primary ingredients. Upon inducing crosslinking between polymer chains, the thermal stability shows a notable enhancement as it occurs at higher ranges with a lower weight reduction. In other words, the second maximum degradations ($T_{max}$) for the networked hydrogels escalate by 20.8 and 55 units, respectively. The first weight-loss step becomes remarkably negligible, indicating the formation of interconnected chains. The crosslinking process establishes permanent covalent bonds among the chains, resulting in a three-dimensional network that induces a more robust polymer structure [34]. Such a network exhibits amended stability facing physicochemical stresses, including thermal treatment and dissolution. Furthermore, the calculated residual mass of the crosslinked hydrogel at the $T_{max}$ = 500 °C provides additional evidence of the improved thermal decomposition resistance following crosslinking, wherein the networked SA/CS hydrogel retains a significantly higher mass fraction of 41.12% (Table 2). Thermal stability for the single-particle hydrogel nanocomposite improves as $T_{max}$ increases to the higher range, demonstrating less destruction.

All hydrogel nanocomposites exhibit two distinct $T_{max}$ values, wherein the first peak corresponds to the thermal decomposition of SA and the subsequent peak is attributed to the degradation of its counterpart. This tendency is also correct for hybrid hydrogel nanocomposites. We observe that the second $T_{max}$ has a minimal change, while the first value has a more pronounced alteration. For example, the $T_{max}$ for the hydrogel nanocomposite containing CNT:GNP (1:3) is increased by 24.8 °C compared to the crosslinked SA/CS hydrogel, although it is increased by 16.3 °C for 3% GNP. Hybrid samples comprising CNT:GNP in ratios of 1:3 and 1:1 exhibit synergistic properties, wherein the planar graphene sheets confer a protective effect by acting as a thermal barrier. Such an improvement is further promoted by the dispersing capability of GNP, which facilitates the uniform distribution of CNT.

### 3.3. Kinetics of Ion Absorbance in Hydrogel Nanocomposites

**Figure 4:**
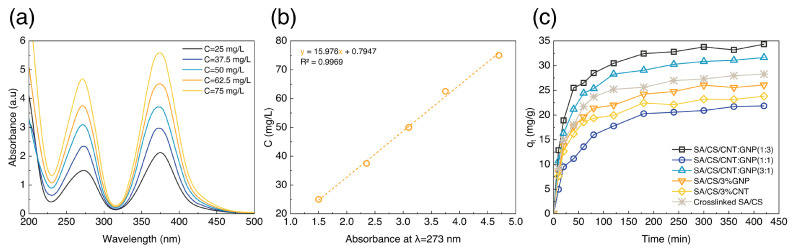
Chromium ion absorbance of the SA/CS hydrogel nanocomposites. (**a**) UV-vis spectra of chromium ion adsorption. (**b**) Calibration graph of UV-vis spectra. (**c**) Ion adsorption capacity at $C_{0}=50\;mg/L$.

By carrying out batch method experiments on the hydrogels for the adsorption of ions, samples were obtained and evaluated using UV spectroscopy. The results of UV spectroscopy for different concentrations of chromium ions in the range of 25–75 mg/L along with its calibration diagram are shown in Figure 4a,b. The wavelength of the maximum adsorption after the formation of the complex with ammonia is about 273 nm. The equation of calibration is y = 15.976x + 0.7947 with a correlation coefficient of R² = 0.9969, stating the best maximum adsorption data. We then determined the maximum concentration of chromium ions ($C_{t}$) adsorbed in the solution, thereby elucidating the adsorptive capacity of the hydrogels with $q_{t}=\frac{C_{0}-C_{t}}{m}\times V$, where $C_{0}$ refers to the initial ion concentration and *V* = 100 mL is the volume of ion solution absorbed by *m* = 2 g of the hydrogel.

As shown in Figure 4c, the hybrid hydrogel nanocomposite containing 3 wt% GNP exhibits maximum adsorption intensity during the initial stage. This rate remains notably at its highest level for all hydrogels up to 40 min, displaying a rapid adsorption process. Despite continued adsorption up to 240 min, the curves for all samples exhibit a plateau, indicating attainment of equilibrium and maximum ion adsorption capacity. In other words, the adsorption sites approach saturation beyond 240 min, resulting in negligible adsorption thereafter. The adsorption capacity of single-particle hydrogel nanocomposites is enhanced owing to the inherent ability of CNT to capture and absorb metal ions [35]. In contrast to GNP, the SA/CS hydrogel nanocomposite containing CNT indicates reduced adsorption capacity due to its relatively lower surface area. This discrepancy can be attributed to the platelet structure of GNP, which provides a greater abundance of active sites on the graphene sheets, thereby facilitating the adsorption of more ions [36].

The adsorption capacity of chromium ions in the hybrid nanocomposites implies a significant enhancement compared to the single-particle nanocomposites. This performance corresponds to the synergistic effect of carbonic nanoparticles with distinct structures (one-dimensional CNT and two-dimensional GNP), a phenomenon that optimizes their dispersion within the hydrogel matrix and increases the surface area. The adsorption capacity of hybrid SA/CS hydrogel nanocomposites shows, in particular, a direct dependence on the GNP content, with a marked 57.2% expansion in capacity observed as the GNP content increases, surpassing that of the pure SA/CS hydrogel.

**Figure 5:**
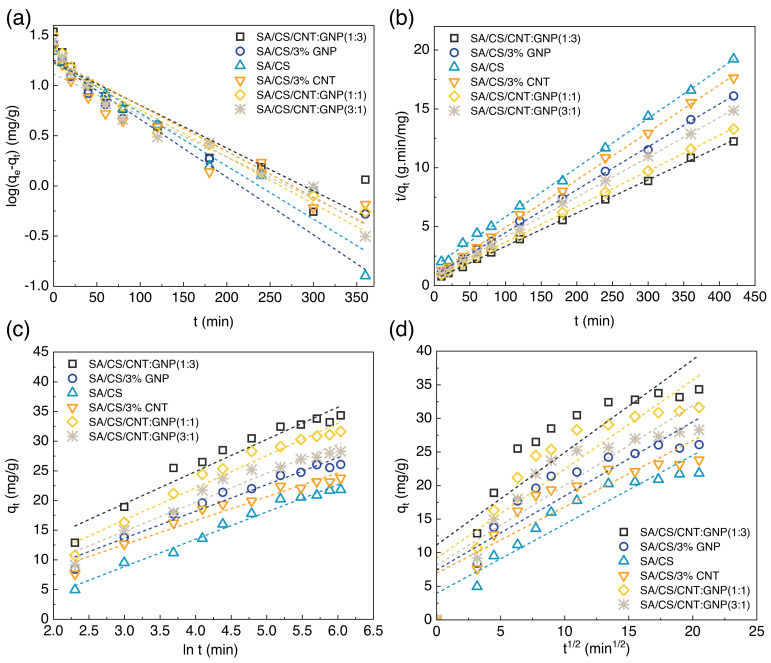
Fitting curves of adsorption in SA/CS hydrogel nanocomposites. (**a**) Pseudo-first-order model. (**b**) Pseudo-second-order model. (**c**) Elovich kinetic model. (**d**) Intraparticle diffusion model.

To corroborate the experimental findings on chromium ion adsorption, we employed theoretical models to unveil the mechanisms involved in the adsorption process. The pseudo-first-order model is commonly utilized to describe sorption kinetics when physical adsorption is the dominant mechanism governing the rate of sorption, as it adequately captures the initial rapid adsorption phase [37]. The differential equation of this kinetic model is expressed as $\frac{dq_{e}}{dt}=k_{1}(q_{e}-q_{t})$, where $q_{e}$ and $q_{t}$ denote the amount of the adsorbent (mg/g) at equilibrium and at a given time (t, min), respectively. $k_{1}$ (min^−1^) is also the rate constant of the pseudo-first-order adsorption. If the boundary conditions are applied from $t=0,\;q_{t}=0$ to $t=t,\;q_{t}=q_{t}$, the integrated rate law for a pseudo-first-order kinetic is derived as $\log\left(\frac{q_{e}}{q_{e}-q_{t}}\right)=\frac{k_{1}}{2.303}t$. The linearized or Lagergren equation of this model is expressed as $\log\left(q_{e}-q_{t}\right)=\frac{log(q_{e}-k_{1})}{2.303}t$. The slope ($k_{1}$) and intercept ($q_{e}$) of the plotted $\log\left(q_{e}-q_{t}\right)$, over time, generates a linear equation that can be fitted to the experimental data (Figure 5a). The constant values of the adsorption rate and correlation coefficients (R^2^) are tabulated in Table 3. As seen, the correlation coefficients indicate a poor fit between the model and the experimental data, revealing the inadequacy of the model to accurately capture the kinetics of chromium ion adsorption by SA/CS hydrogels and their nanocomposites.

Given that the adsorption process involves the formation of covalent bonds through the sharing or exchange of electron density between the adsorbent and the adsorbate, the pseudo-second-order model was appropriately employed to describe this mechanism, as it accommodates the chemical interactions driving the adsorption kinetics [38]. In this model, the differential form is presented as $\frac{dq_{t}}{dt}=k_{2}(q_{e}-q_{t}{)}^{2}$, where $k_{2}$ (g mg^−1^ min^−1^) is the adsorption rate constant. Noting the boundary conditions in this equation gives the integrating equation as $\frac{1}{\left(q_{e}-q_{t}\right)}=\frac{1}{q_{e}}+k_{2}t$, and the linearized form of the pseudo-second-order is $\frac{t}{q_{t}}=\frac{1}{k_{2}q_{e}^{2}}+\frac{t}{q_{e}}$. The values of $q_{e}$ and $k_{2}$ are respectively determined from the slope and intercept of the linear graph, as stated in the previous model. The fitting kinetics model is shown in Figure 5b. Also, the values of R^2^ and $k_{2}$ of the pseudo-second-order model for chromium ions are tabulated in Table 4. The exceeding R^2^ = 0.99 for all hydrogel samples indicates that the kinetics of chromium ion adsorption aligns with the pseudo-second-order model, thus validating its suitability as a descriptive model. This strong correlation substantiates the applicability of the pseudo-second-order model to elucidate the underlying mechanisms governing chromium ion adsorption by the hydrogel samples. The highest value for the equilibrium adsorption capacity in this kinetic model was obtained for the hybrid sample of CNT:GNP (1:3).

The Elovich model finds application in chemisorption kinetics, particularly on heterogeneous adsorbing surfaces, where its validity has been established in describing the adsorption process [39]. To understand further the chemical adsorption mechanism controlling the ion uptake by hydrogels, we applied the Elovich model to the experimental data. The equation is formulated as $\frac{dq_{t}}{dt}=\alpha exp(-\beta q_{t})$, and its integrated form conforms to $q_{t}=\frac{1}{\beta }\ln\left(\alpha \beta \right)+\frac{1}{\beta }\ln t$, where α (mg g^−1^ min^−1^) is the initial adsorption rate and *β* (g mg^−1^) is the constant related to the surface area and the activation energy for chemical adsorption. Figure 5c and Table 5 indicate the fitting of the Elovich model to the adsorption data of chromium ions.

The pure crosslinked SA/CS hydrogel exhibits the highest correlation coefficient value of 1, indicating a strong adaption with the Elovich model. This result confirms that the adsorption kinetics of chromium ions onto the pure hydrogel can be adequately described by the Elovich model. In contrast, the hydrogel nanocomposites display a remarkable deviation, as evidenced by their lower R^2^ values. This discrepancy implies that the Elovich model is not a suitable fit for chromium ion adsorption in nanocomposite hydrogels because of the multi-step mechanisms involved in the adsorption. Also, the heterogeneity of the surface is perceived as a parameter to fail the Elovich model for justification of the kinetics adsorption, since CNT and GNP are present in the hydrogel nanocomposites. It is known that diverse surface properties may be the root of observing such an anomaly in the experimental data compared to the Elovich model, including the variation of the functional moieties, nanoparticle aggregation, and surface roughness or porosity [40]. In this study, these factors mostly contributed to the obtained results from SA/CS hydrogel nanocomposites, revealing the reasoning of the Elovich model.

Another kinetic model for a porous structure is the intraparticle diffusion. This model is expressed by qt=kit12+C, where $k_{i}$ (mg g^−1^ min^−0.5^) is the diffusion rate constant within the adsorbent particle and C is the constant with respect to the boundary layer effect [41]. This equation suggests a diffusion-controlled process as the adsorption rate is proportional to $t^{1/2}$, i.e., the intraparticle diffusion is the only rate-limiting step. Figure 5d shows the fitting of the intraparticle diffusion model to the experimental data of chromium ion adsorption, along with the calculated data in Table 6, obtained from plotting $q_{t}$ over $t^{1/2}$. The experimental data for the pure hydrogel and nanocomposite systems exhibit non-linear curves, indicating that the intraparticle diffusion kinetic model plays a subordinate role in the adsorption process. Previous findings have related such a behavior to the complex internal structure of the material [40,42]. Adsorption is indeed hindered when structural deficiencies, namely non-uniform particles, pores, and agglomeration, exist. The variation of functional groups also differs in the alignment of the experimental data with the fitted curves. It can, therefore, be inferred that the adsorption of chromium ions onto hydrogels primarily occurs through physical adsorption mechanisms, rather than intraparticle diffusion-controlled processes.

## 4. Conclusions

In this study, we developed a hybrid hydrogel nanocomposite based on CS and SA containing CNT and GNP. The morphology of the hydrogel nanocomposites and the hybrid samples showed a uniform dispersion of nanoparticles throughout the SA/CS hydrogel. This was attributed to the distinct structure of the nanoparticles as one-dimensional GNP sheets which precluded two-dimensional CNT particles from reaggregating. The decomposition temperature of hybrid hydrogel nanocomposites showed an upward shift toward higher temperatures due to the synergistic effect of CNT and GNP on thermal resistance. The ion adsorption of the SA/CS hydrogel with nanoparticles improved due to the trapping of chromium ions into the surface of carbonic nanomaterials. The adsorption kinetic models fitted to the data revealed good alignment between the pseudo-second-order and the experimental data of pure and nanocomposite hydrogels. The Elovich model was also in favor of the latter kinetic model, except for the nanocomposite samples. The discrepancies between the Elovich model predictions and the adsorption capacity of single-particle and hybrid nanocomposites unveiled the intricacy of the adsorption mechanisms, likely influenced by the porosity of the hydrogels, as well as the surface heterogeneity caused by CNT and GNP. The plotted intraparticle diffusion kinetic model for pure and nanocomposite hydrogels failed to show consistency with the experimental data owing to the complex structure of the hydrogels formed with CNT, GNP, functional groups of polymers, and internal porosity of the hydrogel.

## Figures and Tables

**Table 1:** The crystallinity index of the neat polymers and hydrogels.

Sample	Crystallinity Index (%)	Average Crystallite Size (nm)
CS	56.86	19.90
SA	26.11	16.83
SA/CS	36.91	13.26
SA/CS/3%CNT	27.72	16.02
SA/CS/3%GNP	7.96	4.15
SA/CS/CNT:GNP (3:1)	31.00	17.63
SA/CS/CNT:GNP (1:1)	21.44	17.23
SA/CS/CNT:GNP (1:3)	12.63	0.96
$\mathrm{Scherrer}\;\mathrm{equation}:\;D=\frac{K\lambda }{\beta cos\theta }$; *D* denotes the crystallite size (nm), *K* = 0.9 is the Scherrer constant, *β* represents FWHM, *λ* = 1.54056 nm is the X-ray source wavelength, and *θ* is the peak position.		

**Table 2:** The maximum degradation and charcoal residue of neat polymers, SA/CS hydrogel, and SA/CS hydrogel nanocomposites.

Sample	*T*_*max*,1_ (°C)	*T*_*max*,2_ (°C)	Charcoal Residue (%) at 500 °C
SA	81.6	232.4	9.25
CS	89.1	300.8	28.63
Uncrosslinked SA/CS	87.8	289.3, 232.4	18.38
Crosslinked SA/CS	-	344.3, 253.2	41.12
SA/CS/3%CNT	-	331.4, 252.2	44.81
SA/CS/CNT:GNP (3:1)	-	337.9, 256.2	45.48
SA/CS/CNT:GNP (1:1)	-	334.2, 262.3	46.48
SA/CS/CNT:GNP (1:3)	-	335.7, 267.4	46.89
SA/CS/3%GNP	-	333.9, 258.9	46.13

**Table 3:** The calculated kinetic parameters from the pseudo-first-order model.

Sample	Equation	*k*_1_	R^2^
Crosslinked SA/CS	y = −0.0053x + 1.2547	0.0122	0.9572
SA/CS/3%CNT	y = −0.0042x + 2.4210	0.0097	0.9168
SA/CS/3%GNP	y = −0.0058x + 1.2385	0.0133	0.8073
SA/CS/CNT:GNP (3:1)	y = −0.0047x + 1.2200	0.0108	0.9494
SA/CS/CNT:GNP (1:1)	y = −0.0046x + 1.2567	0.0106	0.9554
SA/CS/CNT:GNP (1:3)	y = −0.0043x + 1.2310	0.0099	0.8817

**Table 4:** Computed kinetic parameters from the pseudo-second-order model.

Sample	Equation	*q_e_*	*k*_2_	R^2^
Crosslinked SA/CS	y = 0.0418x + 1.6441	23.92	0.0011	0.9989
SA/CS/3%CNT	y = 0.0403x + 0.9010	24.81	0.0018	0.9992
SA/CS/3%GNP	y = 0.0364x + 0.8457	27.47	0.0016	0.9994
SA/CS/CNT:GNP (3:1)	y = 0.0338x + 0.7614	29.59	0.0015	0.9996
SA/CS/CNT:GNP (1:1)	y = 0.0302x + 0.6672	33.11	0.0014	0.9999
SA/CS/CNT:GNP (1:3)	y = 0.0282x + 0.5147	35.46	0.0015	0.9996

**Table 5:** The calculated kinetic parameters from the Elovich model.

Sample	Equation	*β*	*α*	R^2^
Crosslinked SA/CS	y = 4.6025x − 4.9405	0.217	1.577	0.9814
SA/CS/3%CNT	y = 4.0304x + 0.4868	0.248	4.549	0.9504
SA/CS/3%GNP	y = 4.5267x + 0.0674	0.221	4.593	0.9624
SA/CS/CNT:GNP (3:1)	y = 4.8981x + 0.1197	0.204	5.023	0.9519
SA/CS/CNT:GNP (1:1)	y = 5.4300x + 0.4269	0.184	5.879	0.9609
SA/CS/CNT:GNP (1:3)	y = 5.4161x + 3.2273	0.185	9.820	0.9439

**Table 6:** The computed kinetic parameters from the intraparticle diffusion model.

Sample	Equation	*k_i_*	*C*	R^2^
Crosslinked SA/CS	y = 1.0275x + 3.9607	1.0275	3.9607	0.8934
SA/CS/3%CNT	y = 0.9872x + 6.9535	0.9872	6.9535	0.7931
SA/CS/3%GNP	y = 1.1019x + 7.4424	1.1019	7.4424	0.8080
SA/CS/CNT:GNP (3:1)	y = 1.1894x + 8.1273	1.1894	8.1273	0.7982
SA/CS/CNT:GNP (1:1)	y = 1.3270x + 9.1904	1.3270	9.1904	0.8014
SA/CS/CNT:GNP (1:3)	y = 1.3787x + 11.1500	1.3787	11.1500	0.7635

## Data Availability

No additional data are available.
